# 1α,25-dihydroxyvitamin D_3_ protects retinal ganglion cells in glaucomatous mice

**DOI:** 10.1186/s12974-021-02263-3

**Published:** 2021-09-16

**Authors:** Francesca Lazzara, Rosario Amato, Chiara Bianca Maria Platania, Federica Conti, Tsung-Han Chou, Vittorio Porciatti, Filippo Drago, Claudio Bucolo

**Affiliations:** 1 Department of Biomedical and Biotechnological Sciences, Section of Pharmacology, School of Medicine, University of Catania, Catania, Italy; 2 Bascom Palmer Eye Institute, Miller School of Medicine, University of Miami, Miami, FL USA; 3 Department of Biology, University of Pisa, Pisa, Italy; 4 Center for Research in Ocular Pharmacology — CERFO, University of Catania, Catania, Italy

**Keywords:** Inflammation, Cytokines, Glaucoma, Calcitriol, Retina, Vitamin D

## Abstract

**Background:**

Glaucoma is an optic neuropathy characterized by loss of function and death of retinal ganglion cells (RGCs), leading to irreversible vision loss. Neuroinflammation is recognized as one of the causes of glaucoma, and currently no treatment is addressing this mechanism. We aimed to investigate the anti-inflammatory and neuroprotective effects of 1,25(OH)_2_D_3_ (1α,25-dihydroxyvitamin D_3_, calcitriol), in a genetic model of age-related glaucomatous neurodegeneration (DBA/2J mice).

**Methods:**

DBA/2J mice were randomized to 1,25(OH)_2_D_3_ or vehicle treatment groups. Pattern electroretinogram, flash electroretinogram, and intraocular pressure were recorded weekly. Immunostaining for RBPMS, Iba-1, and GFAP was carried out on retinal flat mounts to assess retinal ganglion cell density and quantify microglial and astrocyte activation, respectively. Molecular biology analyses were carried out to evaluate retinal expression of pro-inflammatory cytokines, pNFκB-p65, and neuroprotective factors. Investigators that analysed the data were blind to experimental groups, which were unveiled after graph design and statistical analysis, that were carried out with GraphPad Prism. Several statistical tests and approaches were used: the generalized estimated equations (GEE) analysis, *t*-test, and one-way ANOVA.

**Results:**

DBA/2J mice treated with 1,25(OH)_2_D_3_ for 5 weeks showed improved PERG and FERG amplitudes and reduced RGCs death, compared to vehicle-treated age-matched controls. 1,25(OH)_2_D_3_ treatment decreased microglial and astrocyte activation, as well as expression of inflammatory cytokines and pNF-κB-p65 (*p* < 0.05). Moreover, 1,25(OH)_2_D_3_-treated DBA/2J mice displayed increased mRNA levels of neuroprotective factors (*p* < 0.05), such as BDNF.

**Conclusions:**

1,25(OH)_2_D_3_ protected RGCs preserving retinal function, reducing inflammatory cytokines, and increasing expression of neuroprotective factors. Therefore, 1,25(OH)_2_D_3_ could attenuate the retinal damage in glaucomatous patients and warrants further clinical evaluation for the treatment of optic neuropathies.

## Introduction

Retinal ganglion cell (RGC) degeneration plays a key role in glaucoma and the etiopathogenesis of the disease has not yet been clearly defined. On the other hand, the lack of neurotrophic support and mechanical axonal injury, linked to increased intraocular pressure (IOP), have been reported as detrimental causes of RGCs death [1]. Since damaged RGCs cannot repair or regenerate [2], RGCs loss results in optic neuropathy, optic nerve cupping and then irreversible vision loss [3]. Furthermore, preclinical and clinical studies evidenced that glaucoma can progress even during ocular hypotensive treatment [4–6]; therefore, current glaucoma treatments partially address the mechanisms behind RGCs degeneration and death [7]. A primary contribution in the multifactorial aetiology of glaucoma has been attributed to inflammation, which impacts RGCs function, along with mechanical stress due to high IOP levels [8]. These detrimental inflammatory processes occur at different stages of glaucoma and involve RGCs and retinal resident glial cells (i.e., astrocytes and microglia) [9]. Therefore, the progressive RGCs degeneration could potentially be counteracted by the inhibition of neuroinflammation.

Vitamin D_3_ (cholecalciferol) is produced in the skin when 7-dehydrocholesterol is exposed to solar ultraviolet radiation. In the liver cholecalciferol is converted into calcifediol (25-OH-cholecalciferol or 25(OH)D_3_) and in the kidneys into calcitriol (1,25(OH)_2_-cholecalciferol or 1α,25-dihydroxyvitamin D_3_ or 1,25(OH)_2_D_3_) which is the high-affinity ligand to the nuclear receptor vitamin D receptor (VDR) [10, 11]. The active form of vitamin D_3_, 1,25(OH)_2_D_3_, is endowed with anti-inflammatory and immunomodulatory properties. Due to the widespread expression of VDR, several functions have been associated with this receptor, such as the regulation of proliferation and cell differentiation, apoptosis, angiogenesis, and immunomodulation [12], in cancer [13], multiple sclerosis [14], and cardiovascular diseases [15]. In the central nervous system, 1,25(OH)_2_D_3_ and its metabolites have been shown to exert neuroprotective effects [16] in several neurodegenerative disorders [17, 18]. Moreover, 1,25(OH)_2_D_3_ has been reported to attenuate the macrophage production of pro-inflammatory cytokines and chemokines [19], such as interferon (IFN)-γ [20]. Furthermore, 1,25(OH)_2_D_3_ provided neural protection in aged mice through the modulation of neurotrophic factors such as nerve growth factor (NGF) [21] and BDNF [22]. Eye tissues express both VDR and 1,25(OH)_2_D_3_ regulatory enzymes. In fact, in human eye, immunohistochemical staining identified the expression of VDR in the epithelium of the cornea, lens, ciliary body, and retinal pigmented epithelial cells, as well as, ganglion cell layer, and photoreceptors [23]. We hereby aimed to evaluate the neuroprotective and anti-inflammatory effects of 1,25(OH)_2_D_3_ in a DBA/2J mouse model of inherited glaucoma [24]. In particular, the effect of 1,25(OH)_2_D_3_ on the RGCs function was analysed with pattern electroretinogram (PERG), a selective approach to monitor RGCs electrical activity. The light-adapted flash electroretinogram (FERG) was also carried out to test the outer retinal activity. Moreover, we carried out a third-party bioinformatic analysis of involved molecular pathways, and accordingly, we assessed the neuroprotective and anti-inflammatory effects of 1,25(OH)_2_D_3_ in retina of glaucomatous DBA/2J mice.

## Methods

### Animals and experimental design

#### Animals

The DBA/2J mouse strain is a well-established model of spontaneous glaucoma with progressive loss of RGCs and optic disc excavation, which represent the hallmarks of glaucoma [24].

**Fig. 1:**
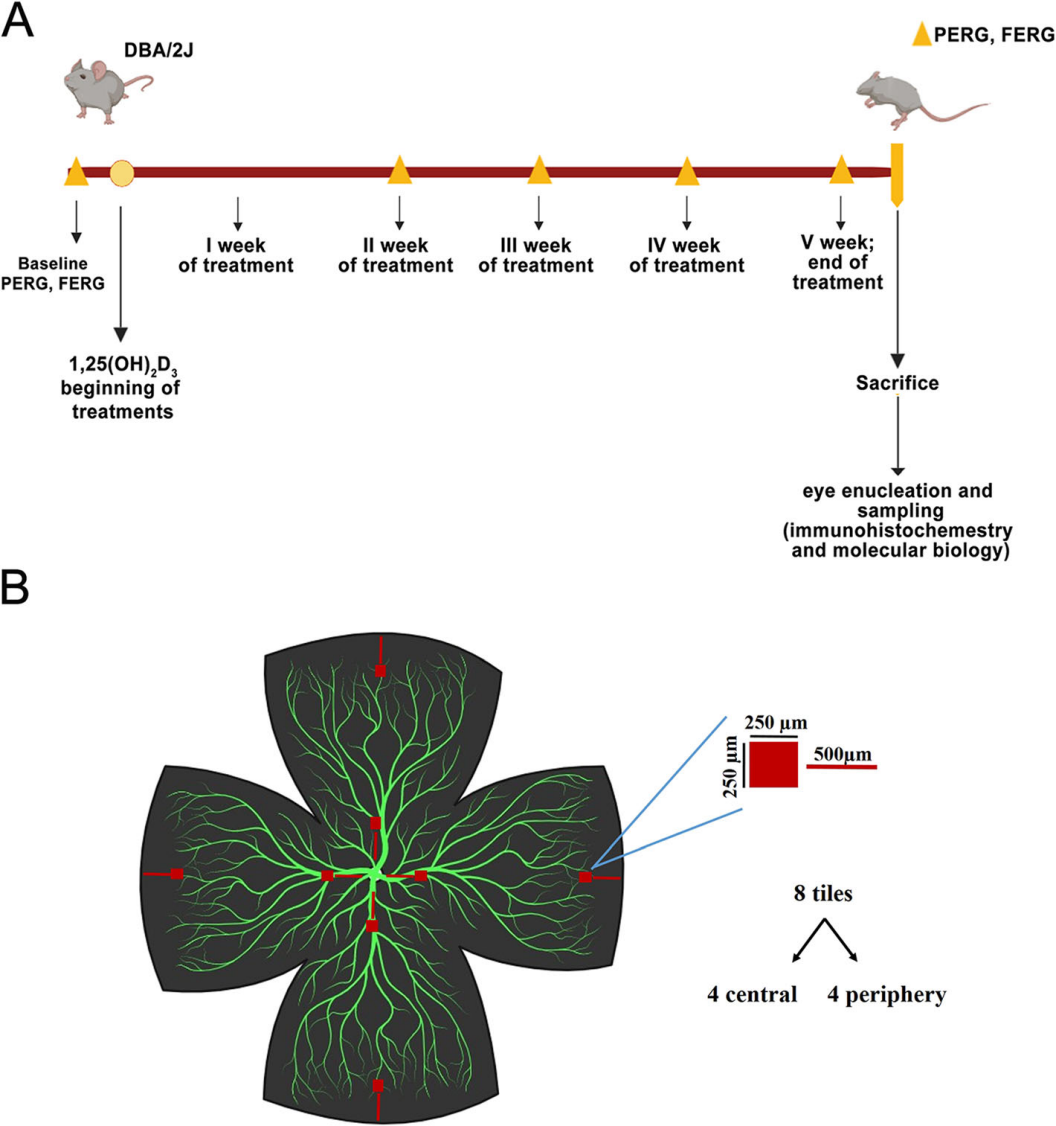
Pharmacological treatment. **A** Experimental design. Baseline PERG and FERG recordings were performed before treatment. The 1,25(OH)_2_D_3_ treatment was carried out, for 5 days per week, for 5 weeks. PERG/FERG recordings were carried out once per week. At the end of 5 weeks, mice were sacrificed, and tissues processed for immunohistochemical staining and molecular biology analyses. **B** Immunohistochemistry images sampling on retinal on whole mount. For each retina, 8 images were acquired, 4 related to central retina and 4 from peripheral retina. Six whole mounts per experimental group were analysed. A total of 48 images per experimental group provided RBPMS+, Iba-1+, and GFAP+ cells quantifications. Both periphery and central images were acquired at 500-μm distance, from whole mount edge and optic disc hole, respectively

DBA/2J female mice of seven months of age were randomly assigned to either the 1,25(OH)_2_D_3_ treatment (*n* = 15) or the vehicle-treated control group (*n* = 15) (https://www.jax.org/strain/000671, Jackson Laboratories, Bar Harbor, ME, USA). All procedures were performed in compliance with the Association for Research in Vision and Ophthalmology (ARVO) statement for use of animals in ophthalmic and vision research. The experimental protocol was approved by the Animal Care and Use Committee of the University of Miami https://umiamihealth.org/bascom-palmer-eye-institute/research/laboratory-research (protocol number 19-088). All mice were housed in a cyclic light environment (12-h light, 50 lux—12-h dark) and fed with a Grain Based Diet (Lab Diet: 500, Opti-diet, PMI Nutrition International, Inc., Brentwood, MO, USA). Baseline physiological recordings of retinal function were carried out by means of the electroretinogram (FERG) and pattern electroretinogram (PERG) before the beginning of the treatment protocol (Fig. 1A). PERG and FERG recordings were carried out in anesthetized mice (PERG and FERG independent records on each eye of the mice, *n* = 2 per mouse, total *n* = 30 per group), accordingly to previous published studies [25]. To randomize the sampling of retinas for histological and molecular biology analyses, at the end of the treatment protocol, 15 mice per group were sacrificed with cervical dislocation, and eyes were collected. In particular, for each experimental group, 30 eye globes from 15 animals, were randomly collected: six eye globes were randomly dissected and processed for immunostaining (*n* = 6 per group; Fig. 1B) and the remaining contralateral six eyes were randomly included with another eye, from different animal of the same experimental group, in a vial to be used for western blot and qPCR analyses.

#### Pharmacological treatments

The 1,25(OH)_2_D_3_ was purchased from Sigma-Aldrich (D1530, St. Louis, MO, USA). The 1,25(OH)_2_D_3_ was dissolved in ethanol 100% and stored at − 20°C. Fifteen mice were treated (i.p.) with 1,25(OH)_2_D_3_ (50 μL in safflower oil) at the dose of 1 μg/kg (which corresponds to the human equivalent dose of 4.86 μg [26], or ~ 200 IU) every day, 5 days per week, for 5 weeks. This dose corresponds to the human recommended daily intake of 1,25(OH)_2_D_3_, i.e., 5 μg, or 200 IU per oral dose (UE regulation N. 1169/2011). The dose was chosen based on previous findings from our lab and other groups [27]. Age-matched control mice were treated (i.p.) with 50 μL of safflower oil (vehicle, *n* = 15).

#### IOP measurement

Mice were anesthetized by an intraperitoneal injection (0.5 mL/kg) of ketamine and xylazine (42.8 mg/mL and 8.6 mg/mL, respectively). Five minutes after the induction of anaesthesia, IOP was measured with an induction–impact tonometer (TonoLab, Colonial Medical Supply, Franconia, NH, USA). The optical axis of the eye was aligned to the probe tip of the tonometer, distancing it from the cornea 1 to 2 mm. An average of five consecutive readings was considered as a measure of the IOP. The impact of the TonoLab probe on the cornea is minimal and does not cause either corneal damage or progressive changes in IOP, even after many repeated readings.

#### Pattern electroretinogram (PERG)

Pattern electroretinogram (PERG) recordings were carried out, once per week, to assess RGCs function with time. As previously described [28], anesthetized mice were transferred onto a heating plate with the mouse superior incisor teeth hooked to a bite bar; mouse head was gently restrained with head holders. The body was kept at a constant temperature (37.0 °C) with a feedback-controlled heating pad (TCAT-2LV, Physitemp Instruments, Inc., Clifton, NJ, USA). A small drop of balanced saline was topically applied to prevent corneal dryness, during PERG recordings. The PERG was recorded simultaneously from each eye using a commercially available instrument (Jorvec Corp., Miami, FL, USA), through a single subcutaneous electrode positioned in the snout. Visual stimuli consisted of black-white horizontal bars (pattern) generated on LED tablets, which were presented independently to each eye at 10 cm distance (56° vertical × 63° horizontal field; spatial frequency, 0.05 cycles/degree; 98% contrast; 800 cd/m^2^ mean luminance; right-eye reversal, 0.992 Hz; left-eye reversal, 0.984 Hz). Electrical signals recorded from the common snout electrode were averaged (> 1110 epochs), and PERG responses from each eye were isolated by averaging at stimulus-specific synchrony. PERG waveforms consisted of a positive wave (defined as P1) followed by a slower negative wave with a broad trough (defined as N2), both automatically detected by the Jorvec software. Therefore, each waveform was analysed by measuring the peak-to-trough (P1-N2) amplitude, which is defined as PERG amplitude; while, the time-to-peak of the P1 wave, is defined as PERG latency [28]. The PERG analysis, regarding left and right eyes, was performed independently because the development of glaucoma in the DBA/2J mice is asymmetrical [29], the same as in humans [30].

#### Flash electroretinogram (FERG)

The PERG recording session was immediately followed by the analysis of the light-adapted flash ERG, to investigate the corresponding activity of the outer retinal neurons. Each mouse was gently transferred in a custom-made restrainer, with feedback-controlled body temperature. The FERG response was measured by means of two electrodes (0.25-mm diameter silver wire [World Precision Instruments, Sarasota, FL] configured to a semi-circular loop of 2-mm radius) gently placed on the corneal surface by mean of micromanipulators, which avoid interference with the visual field. A subcutaneous stainless-steel needle (Grass, West Warwick, RI), inserted into the scalp midline, was used as a reference electrode. The ground electrode was a subcutaneous stainless-steel needle placed at the root of the tail. Uniform stimuli, for FERG recordings, consisted of strobe flash stimuli of 20 cd/m^2^ per second superimposed on a steady background light (12 cd/m^2^), and presented within a Ganzfeld bowl. Three consecutive responses to 30 flashes, for each eye, were recorded and averaged; the FERG waveforms were analysed with SigmaPlot version 11 software (Systat Software Inc., San Jose, CA) to identify the major positive and negative waves and calculate the sum of their absolute values (peak-to-trough amplitude).

### Immunostaining and immunofluorescence analyses

Mice were sacrificed by cervical dislocation and eyeballs were enucleated. Six fixed retinas out of 30 retinas per experimental group were used for immunostaining. Specifically, at the end of treatments (Fig. 1B), six random eye globes per group were fixed in 4% w/v paraformaldehyde in phosphate buffer saline 0.1 M pH 7.4 (PBS) for 2 h at room temperature. Each contralateral eyeball was used for molecular biology analyses. Then, eye globes were washed with PBS and stored at 4 °C in 30% w/v PBS sucrose solution. Each retina was isolated, rinsed with PBS, and incubated with primary antibodies diluted in PBS containing 2% v/v Triton X-100 and 5% v/v FBS. The following primary antibodies were used for the retinal immunohistochemistry study: RNA Binding Protein, mRNA Processing Factor (RBPMS, #1832 Phosphosolutions, Aurora, CO, USA — dilution 1:500); the ionized calcium-binding adaptor molecule 1 (Iba-1, Fujifilm Wako Chemicals USA Corp., Richmond, VA, USA — dilution 1:1000); and the glial fibrillary acidic protein (GFAP, Cell Signaling Technology #12389, MA, USA — dilution 1:1000). After 48 h of incubation with primary antibodies at 4 °C, retinas were washed with PBS and incubated with appropriated secondary antibodies (1:200) (Alexa Fluor 488 goat anti-rabbit; Alexa Fluor 514 goat anti-mouse; Alexa Fluor 633 goat anti-guinea pig; Invitrogen, ThermoFisher Scientific, Carlsbad, CA, USA) for 48 h at 4 °C. Finally, retinas were rinsed with PBS once again, flat mounted on polarized glass slides and cover slipped in a fluorescence mounting medium with DAPI (Vector Laboratories, cat. n. H-1200). Images were acquired using the Leica TCS SP5 (Leica Microsystems, Wetzlar, Germany) confocal microscope. Four radial opposite tiles were analysed in both the central (500 μm far from the optic nerve head) and the peripheral (500 μm far from the peripheral edge) portions of each retina (Fig. 1B). The z-stack scanning (sampling rate 1 μm) in each tile was carried out to analyse both the ganglion cells and inner plexiform layers (about 50 thickness).

All the images were processed using the software Leica LAS-X to obtain z-stack maximum projections and multichannel images. RGCs and active microglia densities were calculated respectively as the number of RBPMS and Iba-1 positive cells normalized to the scanned area. Astrocytes activation was evaluated by quantification of mean grey levels of GFAP staining density, following the normalization for the image background, by means of using ImageJ software (provided in the public domain by the National Institutes of Health, available online: http://rsbweb.nih.gov/ij/download.html).

### RNA extraction, cDNA synthesis, and quantitative real-time PCR

Mice were sacrificed by cervical dislocation and eyeballs were enucleated. Twelve retinas out of 30 retinas per experimental group, were used for qPCR analyses; in particular, retinas were dissected from each eye, then two retinas from different mice of the same experimental group were pooled in a vial, and homogenized (*n* = 6 independent retinal samples per experimental group). Total RNA was extracted, purified, and suspended in RNase-free water using TRIzol reagent (Invitrogen, Life Technologies, Carlsbad, CA, USA). The A260/A280 ratio of the optical density of RNA samples (measured with Nanodrop spectrophotometer ND-1000, Thermofisher) was 1.95–2.01, confirming RNA purity. cDNA was synthesized from 1 μg RNA with a reverse transcription kit (SuperScript™ II Reverse Transcriptase, Invitrogen, ThermoFisher Scientific, Carlsbad, CA, USA). Real-time RT-PCR was performed with the Rotor-Gene Q using Qiagen QuantiNova SYBR Green Real-Time PCR Kit. The amplification reaction mix included 1 μL (100 ng) of cDNA. Forty-five amplification cycles were carried out for each sample, in triplicate. Melting curve analysis confirmed the specificity of the amplified products. Results were analysed with the 2^−ΔΔCt^ method and expressed as fold change vs. control. Analysed genes were normalized to 18S mRNA levels, a constitutively expressed gene encoding for ribosomal RNA. Primers were purchased from Eurofin Genomics (Milan, Italy). The qPCR analyses adhered to the MIQE guidelines [31]. Primers are listed in Table 1.

**Table 1:** Primers used for real-time polymerase chain reaction (PCR) amplification

Genes	Primer murine sequences
18S	F: 5′-GTTCCGACCATAAACGATGCC-3′; R: 5′-TGGTGGTGCCCTTCCGTCAAT-3′
BDNF	F: 5′-GTTCGAGAGGTCTGACGACG-3′; R: 5′-AGTCCGCGTCCTTATGGTTT-3′
VEGF-A	F: 5′-GCACATAGGAGAGATGAGCTTCC-3′; R: 5′-CTCCGCTCTGAACAAGGCT-3′
PlGF	F: 5′-ATGCTGGTCATGAAGCTGTTCA-3′; R: 5′-GGACTGAATATGTGAGACACCT-3′
IL-6	F: 5′-CCAGAGCTGTGCAGATGAGTA-3′; R: 5′-TGGGTCAGGGGTGGTTATTG-3′
IL-1β	F: 5′-ACATCAGCACCTCACAAGCAGAG-3′; R: 5′-TGGGGAAGGCATTAGAAACAGTC-3′
IFN-γ	F: 5′-TGCATCTTGGCTTTGCAGCTCTTCCTCATG-3′; R: 5′-TGGACCTGTGGGTTGTTGACCTCAAACTTG-3′
CCL-3	F: 5′-TGAATGCCTGAGAGTCTTGG-3′; R 5′-TTGGCAGCAAACAGCTTATC-3′

### NF-κB western blotting

Mice were sacrificed by cervical dislocation and eyeballs were enucleated. Twelve retinas out of 30 retinas per experimental group, were used for western blot analyses; in particular, retinas were dissected from each eye, then two retinas from different mice of the same experimental group were pooled in a vial and homogenized (*n* = 6 independent retinal samples per experimental group). To assess the activation of the inflammatory pathway in the retina of DBA/2J mice, we analysed the phosphorylation of nuclear factor kappa-B (NF-κB) by western blotting (pNF-κB p65). Eyes were harvested, and retina collected and then homogenized in RIPA buffer containing a protease and phosphatase inhibitors cocktail (Sigma-Aldrich, St. Louis, MO). The protein concentrations of retinal homogenates were quantified with the BCA Assay Kit (Pierce™ BCA Protein Assay Kit, Invitrogen, Life Technologies, Carlsbad, USA). Equal amounts of protein samples (40 μg) were added to the Laemmli buffer (Bio-Rad), boiled at 95 °C for 5 min, loaded on 4–15% Mini-Protean TGX precast gels (Bio-Rad), then subjected to electrophoresis. The separated proteins were transferred onto a 0.2-μm polyvinylidene difluoride membranes (PVDF, Bio-Rad). Membranes were blocked in 5% milk in 1× Tris-buffered saline + Tween (TBST) for 1 h at room temperature. The blocked membranes were incubated with primary antibodies for phospho-NF-κB p65 (Ser536; mouse mAb #3036 Cell Signaling Technology, MA, USA, 1:1000), NF-κB p65 (XP® Rabbit mAb #8242 Cell Signaling Technology, MA, USA, 1:1000), and GAPDH (AB2302 Millipore, Burlington, MA, USA, 1:2000), overnight at 4 °C. Membranes were washed three times for 15 min and then incubated with secondary antibodies (1:10000, ECL anti-mouse, NA931; ECL anti-rabbit NA934, GE Healthcare, IL, USA) for 1 h at room temperature. Membranes were washed three times for 15 min and enhanced chemiluminescence (SuperSignal™ West Pico PLUS Chemiluminescent Substrate, Thermo Fisher Scientific, Carlsbad, CA, USA) solution was used for immunodetection. The relative density of the protein bands was normalized to the levels of GAPDH. The immunoblot bands intensities were quantified using ImageJ software for gel densitometry (provided in the public domain by the National Institutes of Health, available online: http://rsbweb.nih.gov/ij/download.html)

### Gene network analysis

GEO2R analysis of GSE26299 (publicly deposited: Gene expression profiling in DBA/2J glaucoma) was carried out on GEOdataset (https://www.ncbi.nlm.nih.gov/geo/geo2r/) [32]. We retrieved gene expression profiles, from GSE26299, of genes encoding for VDR, BDNF, VEGF-A, PlGF (PGF), IL-6, IL-1β, CCL-3, IFN-γ, and p-65 NF-κB (RELA). This analysis was focused based on current literature data about 1,25(OH)_2_D_3_ modulation. The bioinformatic analysis was carried out both on retina and optic nerve head (ONH) samples from glaucomatous DBA/2J mice (pre-glaucoma, early, moderate and severe glaucoma), and control (D2-Gpnmb+) mice. The focused set of genes was found to be significantly differentially expressed only in ONH of DBA2J mice with severe glaucoma, compared to D2-Gpnmb+. Enrichment analysis on these genes was carried out with GENEMANIA app of cytoscape [33]. The gene/co-expression network generated by GENEMANIA was visualized and analysed with cytoscape 3.7.1. Centrality metrics network analysis was carried out: average shortest path (proportional to node dimension), clustering coefficient (temperature colour scale), and edge betweenness (proportional to edge thickness).

### Statistical analysis

Investigators that analysed the data were blind to experimental groups, which were unveiled after graph design and statistical analysis. In our study, data analysis complied to the recommendations on experimental design and analysis in pharmacology [34]. All data were expressed as mean ± SD.

Assessment of normal distribution of data was carried out with the Shapiro-Wilk test. Levene’s test was used to assess the homogeneity of variance between groups. Statistical significance was assessed by unpaired two-tailed t-test for comparison between two groups. One-way ANOVA with Tukey post hoc test, for multiple comparisons. Post hoc tests were carried out only if F had a *p* < 0.05, and no significant variance inhomogeneity was found within analysed groups. Differences were considered statistically significant for *p* values < 0.05. Statistical analysis was carried out with GraphPad Prism v.5 (GraphPad Software, La Jolla, CA, USA [RRID:SCR_002798]), the same software was used to design graphs. The statistical analysis of PERG values over time (baseline, 2, 3, 4 and 5 weeks of treatment) was carried out with the generalized estimating equations (GEE) approach, through IBM SPSS statistics Ver. 26. GEE is an unbiased non-parametric method that analyse correlated data within time (longitudinal analysis). Statistical significance of effects was evaluated with Wald chi-square. In the GEE analysis, the PERG and FERG amplitude values were the dependent variables, time of treatment (PERG: baseline, 2, 3, 4, and 5 weeks of treatment) and treatments (1,25(OH)_2_D_3_; vehicle) were the predictor variables. Main effects (time of treatment; treatments) and interaction between them were analysed.

## Results

### Effects of 1,25(OH)_2_D_3_ on RGCs function, outer retinal activity, and IOP

**Fig. 2:**
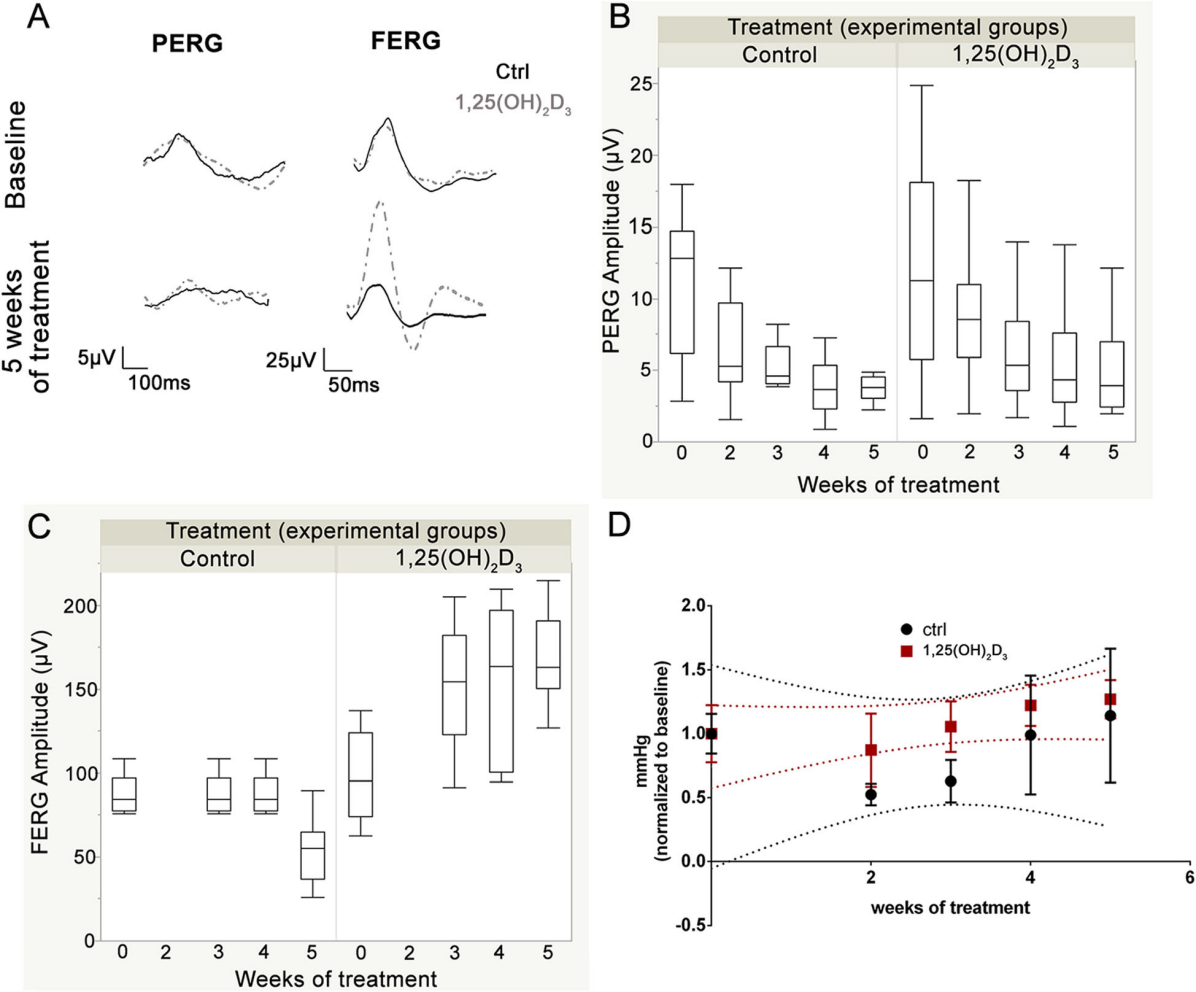
Evaluation of the effects of 1,25(OH)_2_D_3_ treatment on PERG, FERG, and IOP. **A** Representative waveforms of PERG and FERG at baseline and at the end of treatment. Each waveform, deriving from PERG and FERG recordings, was analysed for the peak-to-trough amplitude. **B** (PERG) and **C** (FERG) report the plot of amplitude values vs*.* time of treatment, as regards control DBA/2J and 1,25(OH)_2_D_3_-treated mice. PERG was recorded at baseline and from the 2nd to the 5th week of treatment; FERG was recorded at baseline and from the 3rd week of treatment. **D** IOP values normalized to baseline data, over time. Data were analysed using the GEE statistics to test the effects of time and of treatment; *n* = 30 independent records per experimental group, two eyes per mouse, 15 mice per group.

We analysed the effects of the 1,25(OH)_2_D_3_ on the RGCs and outer retinal functions during 5 weeks of treatment (Fig. 1A) by means of PERG and FERG measurements, respectively (Fig. 2A, representative retinal waveforms). The PERG amplitude (Fig. 2B) decreased in both 1,25(OH)_2_D_3_-treated and control (vehicle-treated) mice, accordingly to the established progression of retinal dysfunction in DBA/2J mice. Nevertheless, in 1,25(OH)_2_D_3_-treated mice PERG amplitude values were significantly (*p* = 0.012) higher than the values recorded in control mice. In fact, GEE statistics revealed a significant effect of treatment (*p* = 0.012) and time of treatment (*p* = 0.0001). However, no statistically significant effect was found between treatment and time of treatment, meaning that 1,25(OH)_2_D_3_ treatment decreased only the rate of progression of retinal dysfunction (*p* = 0.66). In contrast to time-related decline of PERG amplitude values in control DBA/2J mice, the FERG amplitude (Fig. 2C) was constant from baseline (7 month of age) to the 4th week of treatment, and then decreased at the 5th week of treatment. We recorded FERG electroretinograms in all experimental groups at baseline and from the 3rd to the 5th week of treatment. We found that 1,25(OH)2D3 treatment significantly increased the FERG amplitude by about 60%, compared to control mice. GEE statistics confirmed this strong effect of 1,25(OH)_2_D_3_ treatment (*p* = 0.0001) and of time of treatment (*p* = 0.0001). Furthermore, GEE statistical analysis showed that the interaction between treatment and time of treatment was statistically significant (*p* = 0.0001), meaning that 1,25(OH)_2_D_3_ treatment was able to increase activity of the outer retina over time. FERG and PERG amplitudes were not correlated to each other in both controls and 1,25(OH)_2_D_3_-treated mice (*R*^2^=0.01; *p* = 0.6). FERG and PERG latencies did not change with time, in both control mice and 1,25(OH)_2_D_3_-treated mice.

IOP fluctuations were found during treatment, particularly in the control vehicle-treated group compared to 1,25(OH)_2_D_3_-treated mice. During the second and third week of treatment, IOP decreased in the control group, while in 1,25(OH)_2_D_3_-treated mice IOP fluctuation was close to baseline (Fig. 2D). The GEE analysis confirmed that IOP, in all experimental groups, did not impact retinal function. In fact, GEE analysis of FERG and PERG values, over time, was repeated using IOP as a covariate. The time-dependent variation of PERG and FERG, after treatment with 1,25(OH)_2_D_3_, was independent from the IOP covariation, i.e., the IOP-adjusted GEE analysis of FERG and PERG was virtually identical to the non IOP-adjusted GEE analysis described above.

### GSE26299 re-analysis for inflammatory and neurotrophic markers alterations in glaucoma

**Fig. 3:**
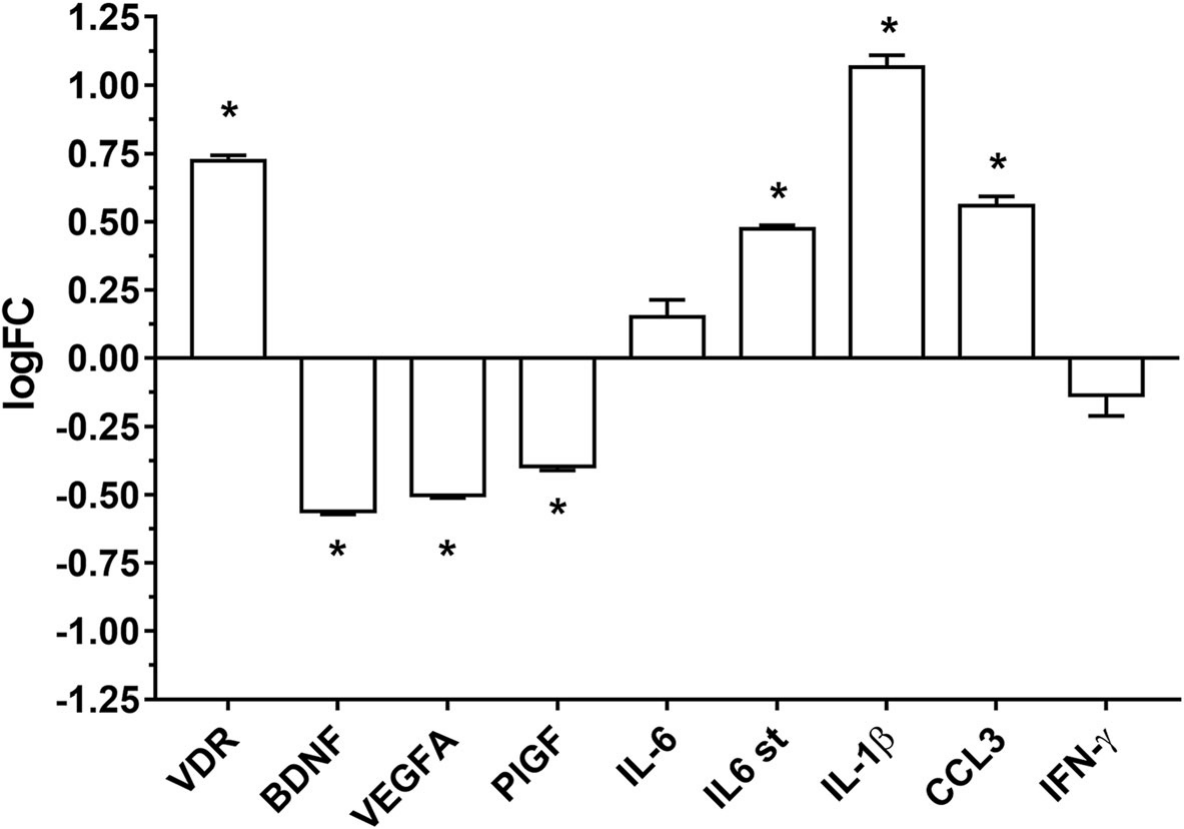
Third-party re-analysis of DBA2J/mice expression profiles deposited on GEOdataset (GSE26299). *p < 0.05 DBA/2J mice with severe glaucoma vs*.* D2-Gpnmb+ control mice. VDR (*p*-value = 8.23e− 04), BDNF (*p*-value = 8.19e− 06), PlGF (*p*-value = 8.35e− 03), IL-6 (not significant), IL-6 signal transducer (IL-6 st) (*p*-value = 2.05e− 03), IL-1β (*p*-value = 1.70e− 02), CCL-3 (*p*-value = 1.70e− 02), and IFN-γ (not significant) (*n* = 10)

DBA/2J expression profile deposited at GEO DataSet (GSE26299) was re-analysed, as third-party material, according to GEO DataSet guidelines (Fig. 3) [32]. Particularly, we retrieved significant statistical differences in gene expression levels in the optic nerve head (ONH) of mice with severe glaucoma, compared to ONH of D2-Gpnmb+ control mice. We focused our analysis on the expression of genes encoding for VDR, BDNF, VEGF-A, PlGF (PGF), IL-6, IL-1β, CCL3, IFN-γ, and p-65 NF-κB (RELA). However, we found statistically significant differential expression for VDR, BDNF, VEGF-A, PlGF, IL-1β, and CCL3. Re-analysis of GSE26299 [32], showed that vitamin D receptor (VDR) expression is increased in ONH of DBA/2J mice (severe glaucoma) compared to control Gpnmb knock-in transgenic mice (D2-Gpnmb+ control). Moreover, BDNF, VEGF-A, and PlGF levels, were found significantly decreased in the ONH of DBA/2J mice with severe glaucoma, compared to control D2-Gpnmb+ mice. Furthermore, re-analysis of GSE26299 showed that DBA/2J mice ONH had higher levels of inflammatory cytokines (IL-1β and CCL3), compared to ONH of D2-Gpnmb+ control mice. IL-6 expression, although not significantly, was increased in ONH of DBA/2J mice; interestingly, we found the IL-6 signal transducer gene (IL-6 st) significantly overexpressed in DBA/2J mice with severe glaucoma. Furthermore, IFN-γ was found to be downregulated, but not significantly, in ONH of DBA/2J mice with severe glaucoma, compared to control mice.

### Effect of 1,25(OH)_2_D_3_ on the RGCs density

**Fig. 4:**
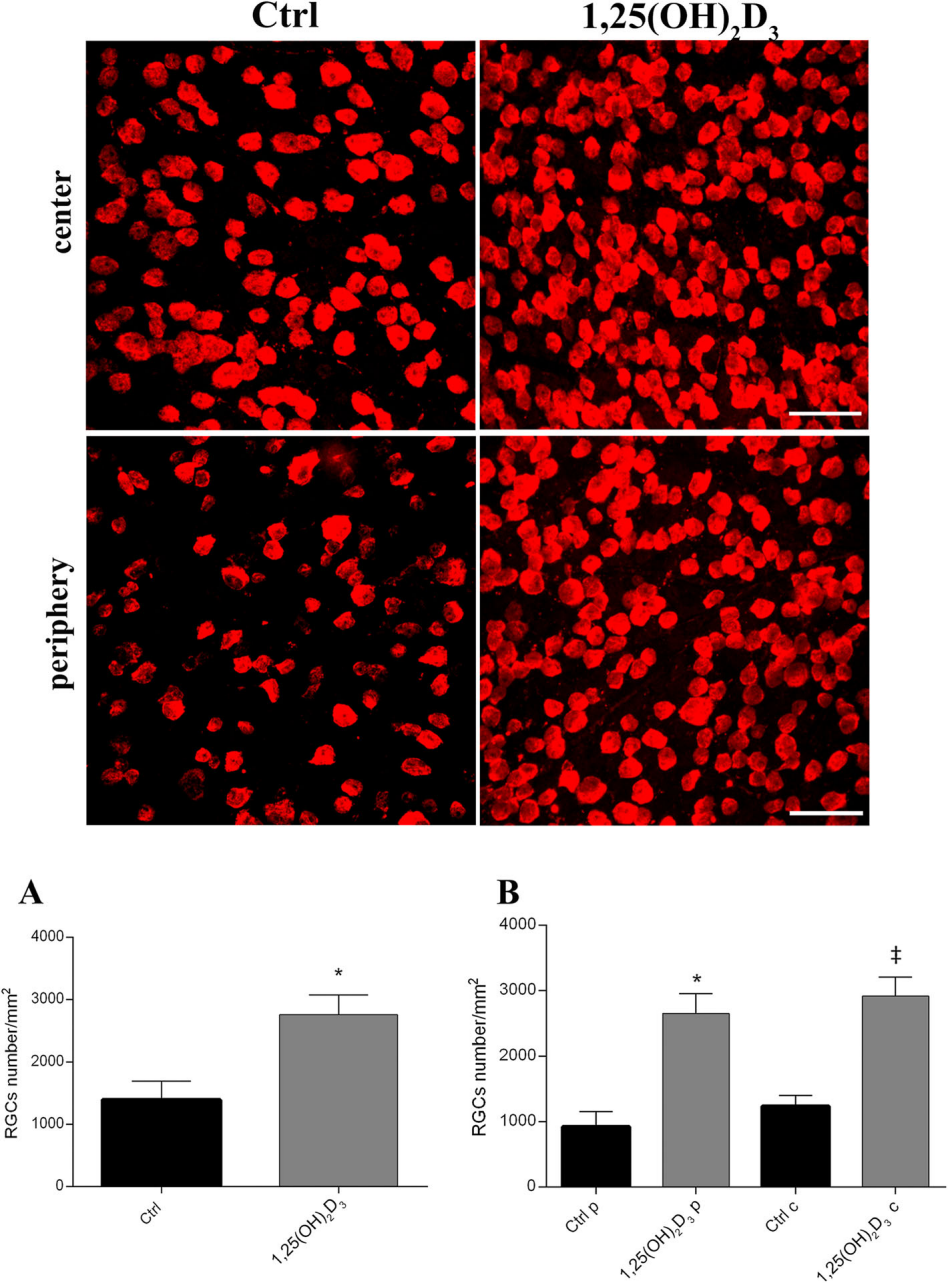
RGCs staining in flat-mount retinas. Upper panel shows representative images of Rbpms^+^ RGCs in whole-mount retinas of control (vehicle-treated) and 1,25(OH)_2_D_3_-treated mice. Scale bar corresponds to 50 μm. RGCs density was significantly higher in 1,25(OH)_2_D_3_-treated mice, compared to the control group. Quantification was carried out on whole retina (**A**) and also on both central and peripheral areas of retina (**B**). Data were plotted as mean ± SD (*n* = 6 flat-mount retinas for each experimental group, *n* = 48 images per experimental group). **A** *p = 0.036 vs. Ctrl; *t*-test two-tailed. **B** *p = 0.0011 vs*.* Ctrl periphery (Ctrl p) ^‡^*p* = 0.0011 vs. Ctrl centre (Ctrl c), one-way ANOVA with Tukey post hoc test for multiple comparison

**Fig. 5:**
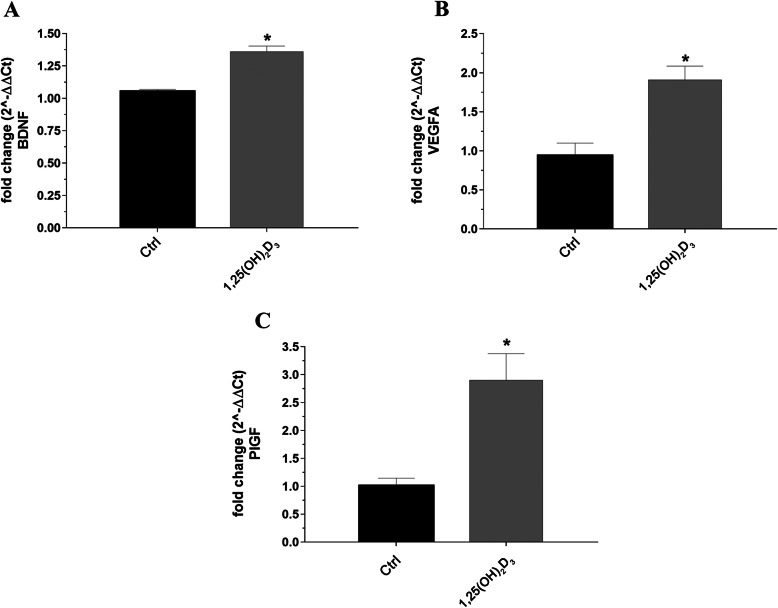
The 1,25(OH)_2_D_3_ and retinal neurotrophic factors. The 1,25(OH)_2_D_3_ increased retinal expression of neuroprotective factors in DBA/2J mice: BDNF (**A**), VEGF-A (**B**), and PlGF (**C**) mRNA expression. Retinal samples of 1,25(OH)_2_D_3_-treated and control mice were collected at the end of treatment. Values represent the mRNA fold changes relative to 18S, a housekeeping gene. Each bar represents the mean value ± SD (*n* = 6 for each experimental group, two pooled retinas per independent sample, each run in triplicate). The 1,25(OH)_2_D_3_ vs*.* control *p* = 0.0006 for BDNF, *p* = 0.0069 for VEGF-A, *p* = 0.0088 for PlGF (unpaired and two-tailed *t*-test)

The 1,25(OH)_2_D_3_ treatment reduced the loss of RGCs compared to the control group and this effect was observed also in the peripheral and in the central areas of the retina. RGC immunostaining presented histopathological features typical of cell dysfunction and/or death, i.e., fragmented and discontinuous Rbpms labelling in control retina, compared to the homogeneous staining observed in retinas of 1,25(OH)_2_D_3_-treated mice (Fig. 4). In fact, the average density of RGCs (Rbpms^+^) was significantly (*p* = 0.036) higher (2754 ± 557 per mm^2^) in 1,25(OH)_2_D_3_-treated mice, compared to the vehicle control group (1391 ± 523 per mm^2^), both in the periphery and centre of the retinas (*p* = 0.0011) (Fig. 4A, B). To assess the neuroprotective effects of 1,25(OH)_2_D_3_, we investigated the expression of neurotrophic factors, which were found to be dysregulated also in ONH of DBA/2J mice (third-party bioinformatic analysis, Fig. 3, GSE26299). We analysed BDNF (*p* = 0.0006), VEGF-A (*p* = 0.0069) and PlGF (*p* = 0.0088) mRNA levels in 1,25(OH)_2_D_3_-treated and control DBA/2J mice retina. We found that mRNA levels of these factors were significantly increased in the retina of 1,25(OH)_2_D_3_-treated mice, compared to the control group (Fig. 5).

### Effect of 1,25(OH)_2_D_3_ on the microglial and glial activation

**Fig. 6:**
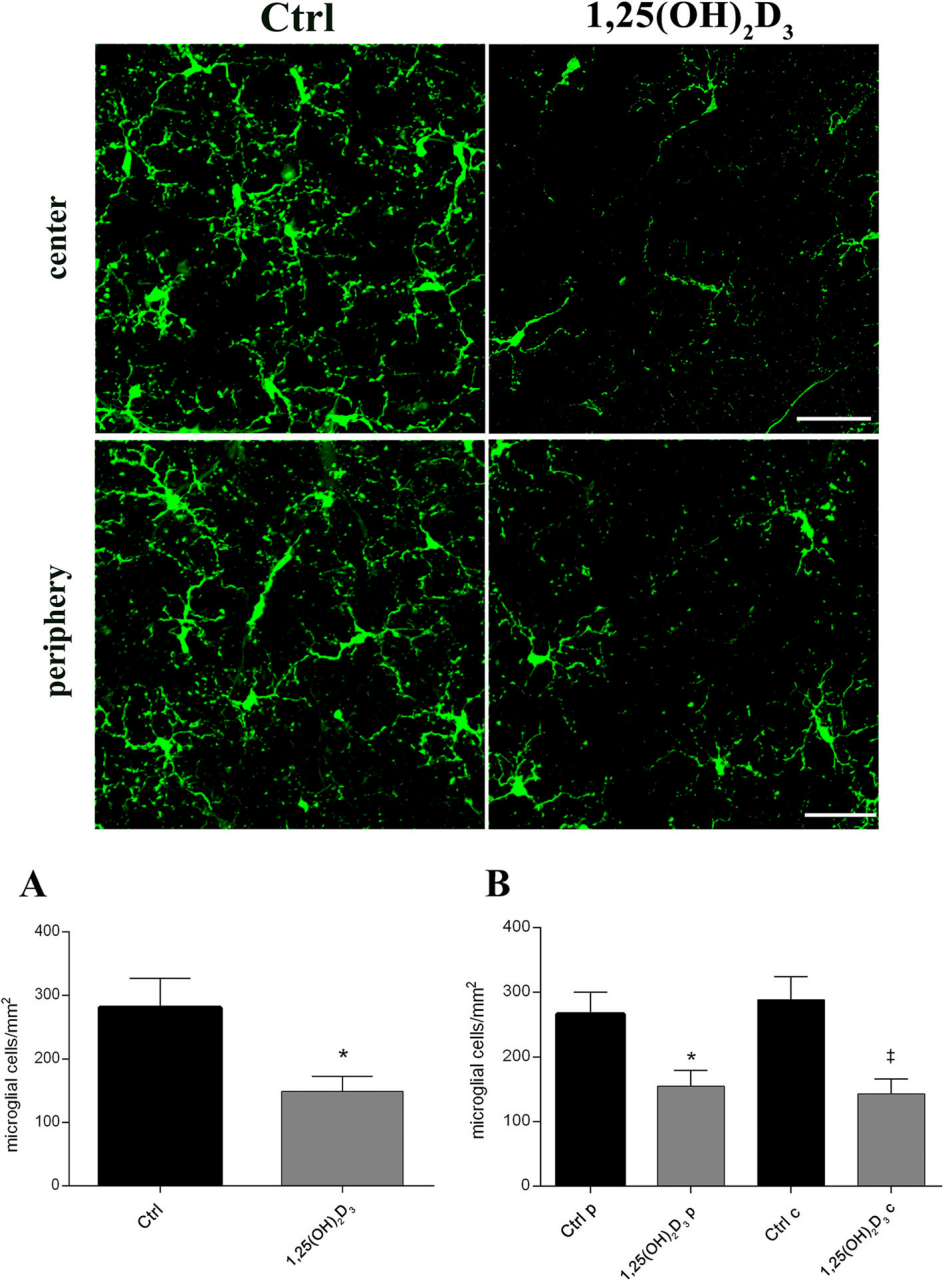
Microglial activation in DBA/2J retina was inhibited after 1,25(OH)_2_D_3_ treatment. Upper panel shows representative images of microglial activation evaluated by immunohistochemistry with the Iba-1 marker in control (vehicle-treated) and 1,25(OH)_2_D_3_-treated groups. Scale bar, 50 μm. The density of activated microglial cells was calculated by counting the number of Iba-1^+^ cells normalized to the scanned area. The quantification was carried out on whole retina (**A**), on both retinal centre and periphery (**B**). Data are plotted as mean ± SD (*n* = 6 flat-mount retinas for each experimental group, *n* = 48 images for each experimental group): **A** *p = 0.0400 vs*.* Ctrl, *t*-test two-tailed. **B** *p = 0.0186 vs. Ctrl periphery (Ctrl p) ^‡^*p* = 0.0186 vs. Ctrl centre (Ctrl c), one-way ANOVA with Tukey post hoc test for multiple comparison

**Fig. 7:**
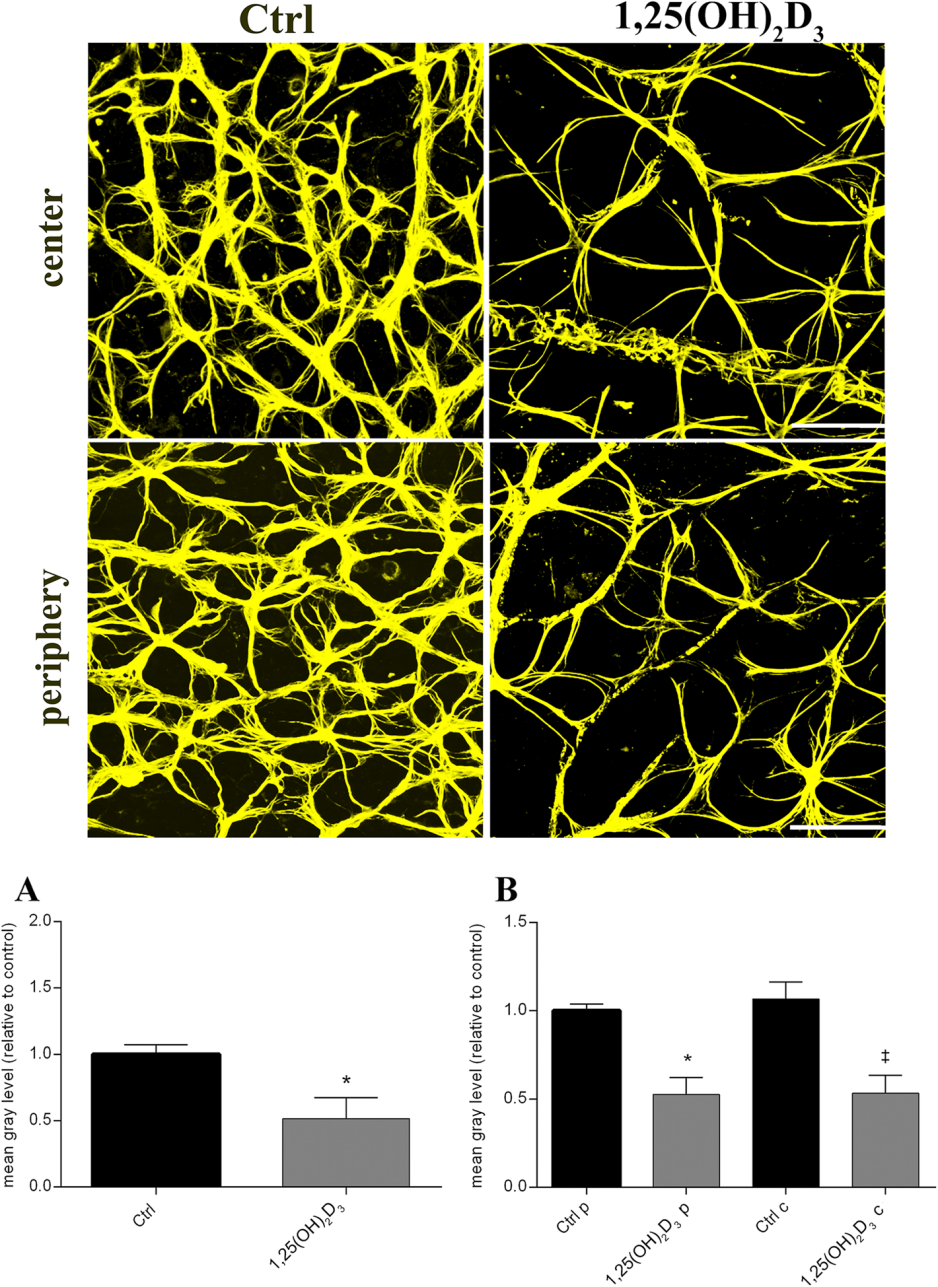
The 1,25(OH)_2_D_3_ treatment reduced reactive astrocytes in DBA/2J mice. Upper panel shows representative images GFAP^+^ astrocytes assessed in flat-mount retina, of control (vehicle-treated) and 1,25(OH)_2_D_3_-treated group. Scale bar, 50 μm. The density of activated astrocytes was quantified, measuring the mean grey levels of GFAP staining density. Quantification was carried out on whole retina (**A**), on both retinal centre and periphery (**B**). Data are plotted as mean ± SD (*n* = 6 flat-mount retinas for each experimental group, *n* = 48 images for each experimental group): **A** *p < 0.0, *p* = 0.0142 vs. Ctrl, *t*-test two-tailed. **B** *p = 0.0029 vs. Ctrl periphery (Ctrl p) ^‡^*p* = 0.0029 vs. Ctrl centre (Ctrl c), one-way ANOVA with Tukey post hoc test for multiple comparisons

We examined the density of Iba-1^+^ cells (Fig. 6), to evaluate the effects of 1,25(OH)_2_D_3_ on retinal microglia activation. Retinas of vehicle-treated DBA/2J mice showed an overall high microglial activation (280.5 ± 79.44 per mm^2^, Fig. 6A), which was not statistically different between the peripheral and central areas of the retina (Fig. 6B). The density of Iba-1^+^ cells was significantly (*p* = 0.0400) decreased in 1,25(OH)_2_D_3_-treated mice (149.1 ± 41.05 per mm^2^, Fig. 6A), compared to control mice, both in peripheral and central areas of the retina (*p* = 0.0186) (Fig. 6B). Cell number and morphology of retinal astrocytes was evaluated in control and 1,25(OH)_2_D_3_-treated flat-mounted retinas, by means of GFAP immunostaining. GFAP staining was higher in DBA/2J control retinas, compared to retinas of 1,25(OH)_2_D_3_-treated DBA/2J mice. In retina of vehicle-treated control mice, reactive astrocytes were hypertrophic both in the peripheral and central retina. The astrocytes in 1,25(OH)_2_D_3_-treated retinas appeared poorly branched and well organized, with a series of delimited processes, radiating from a flattened cell body (Fig. 7). Accordingly, GFAP^+^ astrocyte density was quantified, and the number of reactive astrocytes was significantly (*p* = 0.0142) decreased in the 1,25(OH)_2_D_3_-treated group compared to the control group (Fig. 7A), both in the retinal centre and periphery (*p* = 0.0029) (Fig. 7B).

### The 1,25(OH)_2_D_3_ decreases retinal NF-κB activation and production of inflammatory mediators

**Fig. 8:**
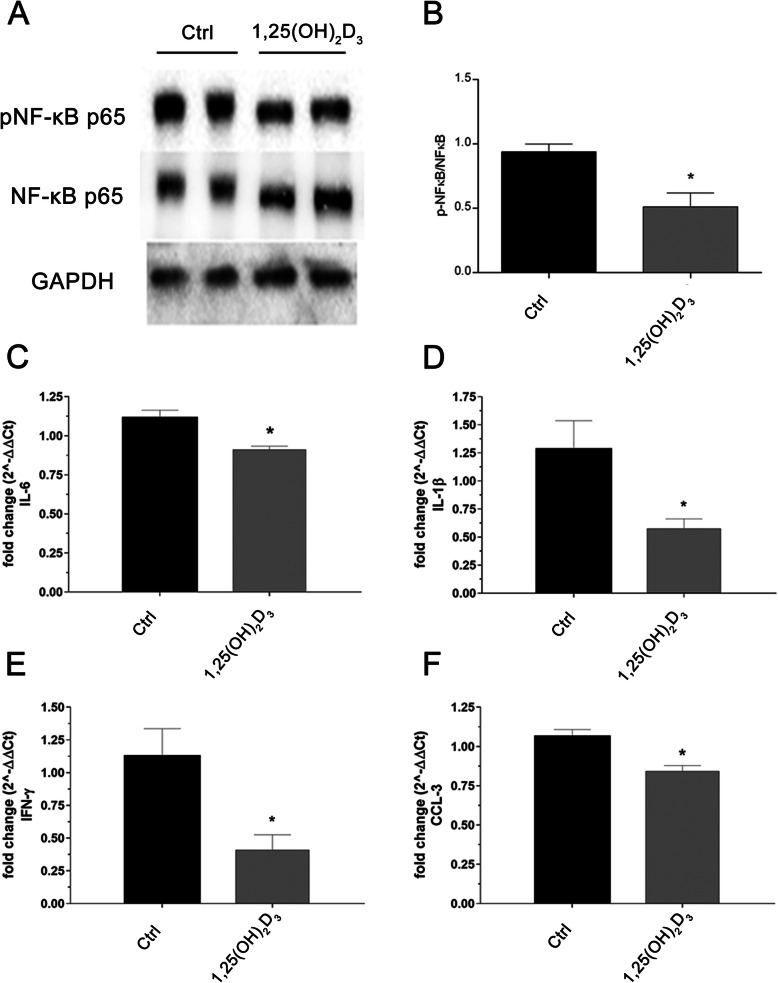
Effect of 1,25(OH)_2_D_3_ on activation of NF-κB and expression of cytokines in DBA/2J mice. **A** Representative blots of phospho-NF-κB-p65, total NF-κB-p65, and GAPDH in DBA/2J retinas (*n* = 6 independent samples for each group, 2 pooled retinas per sample). Expression of phospho-NF-κB-p65 and total NF-κB was quantified using densitometric analysis **B**. Each bar represents the mean value ± SD (*n*= 6). *p = 0.0040 vs*.* control. The 1,25(OH)_2_D_3_ reduced IL-6 (*p = 0.0117, **C**), IL-1β (*p = 0.0412, **D**), IFN-γ (*p = 0.0266, **E**), and CCL-3 (*p = 0.0100, **F**) mRNA expression, compared to control. The mRNA levels were evaluated by qPCR; values represent the mRNA fold changes relative to 18S used as resident control. Each bar represents the mean value ± SD (*n* = 6 independent samples for each group, 2 pooled retinas per sample, each run in triplicate),(unpaired and two-tailed *t*-test)

Iba-1 and GFAP staining confirmed that 1,25(OH)_2_D_3_ treatment significantly decreased the microglial and astrocyte activation in the retina of DBA/2J mice. Furthermore, a series of inflammatory cytokines and the CCL3 chemokine were found to be dysregulated in ONH of DBA/2J mice with severe glaucoma, according to our bioinformatic analysis (Fig. 3, GSE26299). In order to explore the anti-inflammatory effects of 1,25(OH)_2_D_3_, we assessed the activation of the NF-κB pathway in mice retinas by means of western blot analysis of pNF-κB p65 /NF-κB p65 ratio. Immunoblot analysis showed that the level of phosphorylated p65, a component of activated NF-κB, was increased in the vehicle-treated mice retina (Fig. 8). The 1,25(OH)_2_D_3_ treatment significantly (*p* = 0.0040) suppressed the activation of NF-κB. Then, we evaluated the mRNA levels of IL-1β (*p* = 0.0412), IL-6 (*p* = 0.0117), IFN-γ (*p* = 0.0266), and CCL-3 (*p* = 0.0100), and these inflammatory mediators were significantly (*p* < 0.05) downregulated in retinas of 1,25(OH)_2_D_3_-treated DBA/2J mice, compared to levels of vehicle-treated mice (Ctrl) (Fig. 8).

### Post hoc bioinformatic analysis

**Fig. 9:**
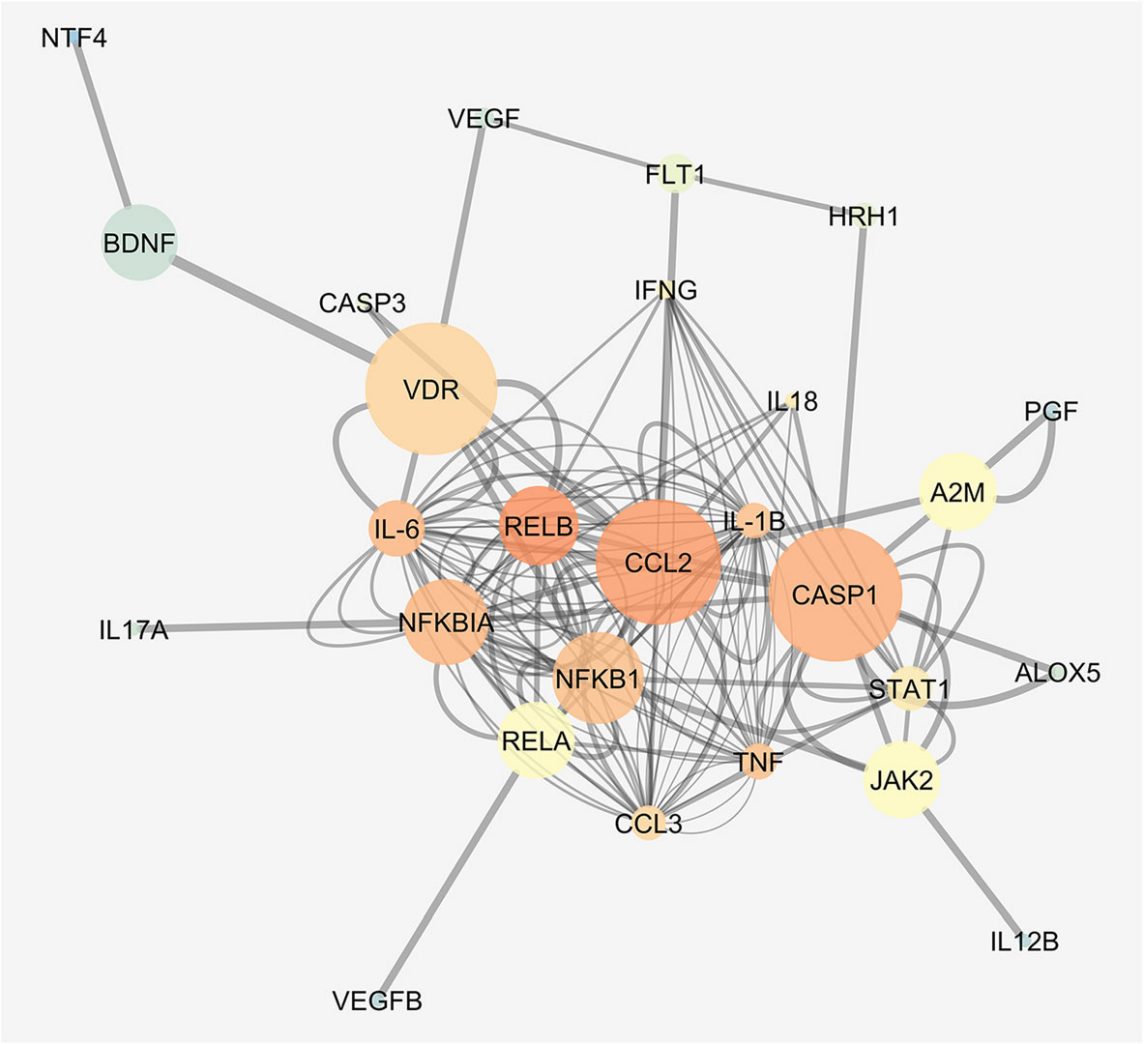
Co-expression network representing 1,25(OH)_2_D_3_ putative mechanism of action. GENEMANIA output was analysed with Cytoscape. Average shortest path (proportional to node dimension), clustering coefficient (temperature colour scale, blue to red for increasing values), and edge betweenness (proportional to edge thickness)

Most of genes, whose expression was found to be modified by 1,25(OH)_2_D_3_ in the retina of 7 months DBA/2J mice in our experimental model were also dysregulated in the ONH of 10 months DBA/2J mice, compared to control D2-Gpnmb+ mice, as shown by our third-party GSE26299 re-analysis (Fig. 3). To gain more information about expression profiles modified by 1,25(OH)_2_D_3_ treatment, we carried out the GENEMANIA bioinformatic analysis of VDR, BDNF, VEGF-A, PlGF (PGF), IL-6, IL-1β, CCL-3, IFN-γ, and p-65 NF-κB (RELA). Figure 9 shows the enriched network (gene/co-expression network), in which genes encoding for BDNF, IFN-γ, CCL-3, IL-6, IL-1β, and p65 NF-κB (RELA) play a central role (high betweenness and shortest path values) in the network stability and node communication efficiency, along with VDR. Gene Ontology (GO) analysis (Table 2) has strengthened, with experimental studies, our initial experimental hypothesis, i.e., 1,25(OH)_2_D_3_ counteracts neuroinflammation and RGCs neurodegeneration in glaucomatous mice.

**Table 2:** GO enrichment analysis of genes modulated in retina of DBA/2J mice, and in ONH of DBA/2J mice (GSE26299). *Q*-value < 0.01

Description	
Positive regulation of cytokine production	
Inflammatory response	
Cytokine receptor binding	
Cytokine activity	
Regulation of inflammatory response	
Positive regulation of response to external stimulus	
Positive regulation of inflammatory response	
Positive regulation of defence response	
Regulation of vitamin metabolic process	
Vitamin D biosynthetic process	
Fat-soluble vitamin biosynthetic process	
Growth factor receptor binding	
Vitamin biosynthetic process	
Response to tumour necrosis factor	
Response to organic cyclic compound	
JAK-STAT cascade	
Vitamin D metabolic process	
Cell-type specific apoptotic process	
Fat-soluble vitamin metabolic process	
Leukocyte differentiation	
Regulation of leukocyte migration	
Angiogenesis	
Regulation of innate immune response	
Vascular endothelial growth factor receptor signalling pathway	
Positive regulation of vascular endothelial growth factor receptor signalling pathway	
Positive regulation of T cell proliferation	
Positive regulation of JAK-STAT cascade	
Regulation of I-κB kinase/NF-κB signalling	
Tyrosine phosphorylation of STAT protein	
Interleukin-17 production	
Regulation of interleukin-17 production	
Leukocyte chemotaxis	
I-κB kinase/NF-κB signalling	
Regulation of vascular endothelial growth factor receptor signalling pathway	
Growth factor activity	
Regulation of angiogenesis	
Cytokine biosynthetic process	
Cytokine metabolic process	
T cell proliferation	
Regulation of vasculature development	
Cellular response to interferon-gamma	

## Discussion

Glaucoma causes vision loss through the degeneration and death of RGCs, even though the mechanisms are not yet fully elucidated. Currently, approved drugs for treatment of glaucoma are aimed at the reduction of intraocular pressure (IOP), which is a well-known risk factor for glaucoma. However, one third of primary open angle glaucomatous (POAG) patients may have optic nerve cupping and visual field defects, regardless of IOP alterations (normotensive glaucoma, NTG). Although a mild beneficial effect of hypotensive drugs has also been demonstrated in NTG patients [4], the glaucomatous RGC degeneration has been shown to persist in POAG as well as in NTG, leading to cell death and irreversible visual impairments. Increasing evidence has recently demonstrated that neuroinflammation and innate immunity may play a leading role in the onset and progression of glaucoma [9]. The 1,25(OH)_2_D_3_ has been shown to have several anti-inflammatory and immunomodulatory functions [12, 20]. Based on these evidences, we investigated the potential protective effects of 1,25(OH)_2_D_3_, in a well-established mouse model of glaucoma, the DBA/2J mice, which is characterized by progressive degeneration of retinal ganglion cells [24].

Retinal electrophysiology (PERG and FERG) was used to assess in vivo retinal function. PERG represents the gold standard tool for the evaluation of RGCs activity. Particularly, PERG is increasingly used as a diagnostic technique for early detection of RGCs dysfunction in suspect glaucoma patients, before the onset of clinical symptoms (visual field defects, and optic nerve cupping) [35–37]. PERG is widely used in preclinical studies, in experimental models of glaucoma, for preclinical evaluation of drug efficacy. In the DBA/2J model of glaucoma, the PERG amplitude values have a negative correlation with the age of DBA/2J mice. This time-dependency of RGCs response is also reflected in increased PERG latency [24, 38]. Accordingly, we registered a time-dependent decline in the activity of the RGCs, measured with PERG, in the vehicle-treated DBA/2J control group. The gradual dysfunction of RGCs, typical of the glaucomatous condition, has been reported to depend on the interplay of several pathological mechanisms, among which the neuroinflammation seems to play a crucial role, although not exclusive [8].

We found that treatment with 1,25(OH)_2_D_3_ resulted in a significant reduction of glaucoma progression rate in DBA/2J mice, as demonstrated by higher PERG amplitude values of 1,25(OH)_2_D_3_-treated mice, during the 5 weeks of treatment. However, the degenerative phenomena of glaucomatous progression were not fully contrasted by 1,25(OH)_2_D_3_, because the decline of PERG amplitude over time was not totally blocked, in agreement with previous findings [39, 40].

The 1,25(OH)_2_D_3_ treatment significantly delayed PERG amplitude loss (RGCs death and dysfunction), and we also found a significant increase of FERG amplitude in 1,25(OH)_2_D_3_-treated mice, compared to control DBA/2J mice. In fact, vehicle-treated control mice exhibited a constant FERG amplitude, as already reported [24], that decreased only at the 5th week of vehicle treatment. FERG signals account mainly for outer retinal function (e.g., photoreceptors, bipolar cells), but also for RGCs activity (photopic negative response) [38, 41]. Moreover, it has been demonstrated that photoreceptors and inner nuclear layer cells of the retina are altered in glaucoma, although the mechanism of outer retinal cells influence on RGCs degeneration has not been fully elucidated. The effect of 1,25(OH)_2_D_3_, on FERG amplitude increase, has possibly influenced the RGCs function through the enhanced input from outer retinal cells, resulting in improved RGCs function (i.e., delayed PERG amplitude loss). However, the GEE analysis did not show a significant correlation between PERG and FERG amplitude values, over the time of treatment. Therefore, the increased function of the first and second order retinal neurons (outer retina, FERG signals) would be one of the several factors influencing RGCs activity. Furthermore, Lee et al. [42], demonstrated that intraperitoneal injections of active form of vitamin D_3_ ameliorated electrical activity of the outer retina, in aged mice.

In glaucoma, the progressive loss of RGCs function has been associated with a progressive increase in IOP. Classically, IOP elevation has been found to correlate with glaucomatous RGC degeneration in ocular hypertensive patients as well as in DBA/2J mice, from 6 months of age. However, changes in the RGC activity measured with PERG do not necessarily indicate a strict dependency between the IOP elevation with RGC dysfunction, because PERG amplitude loss precedes IOP elevation [24, 38]. Furthermore, about 30–40% of glaucoma patients [43] have normal IOP levels, and the current glaucoma therapies are not able to contrast retinal ganglion cells degeneration. This is likely because these interventions decrease only IOP, which is the tip of the iceberg in a complex multifactorial disease. Moreover, IOP cannot be generally considered, at least in preclinical studies, as a drug treatment efficacy endpoint, because IOP fluctuations have been previously recorded within the same day of measurements, also related to gender and age [44].

In the present study, although 1,25(OH)_2_D_3_ treatment did not influence IOP levels in glaucomatous DBA/2J mice (Fig. 2D), some IOP variations were observed weekly in both groups, consistently with typical intra-daily, intra-subjects and inter-subjects IOP variations, usually observed both in mice and glaucoma patients [45]. Particularly, RGCs function improvement in 1,25(OH)_2_D_3_ mice was IOP-independent, as confirmed by GEE analysis, that included IOP as a covariate. In fact, the IOP-adjusted GEE analysis was virtually identical to the non IOP-adjusted GEE analysis, giving the same statistical results.

Along with retinal function evaluation, we carried out immunohistochemical studies and molecular biology analyses to investigate the mechanism of action of 1,25(OH)_2_D_3_ in our experimental model of glaucoma. The 1,25(OH)_2_D_3_ treatment significantly reduced the loss of RGCs in glaucomatous DBA/2J mice, mirroring the retinal functions measured with PERG and FERG recordings. In fact, at the end of the fifth week of treatment, the retina of 1,25(OH)_2_D_3_-treated mice showed a higher RGCs density, compared to the glaucomatous vehicle-treated group, both in peripheral and central retina. RGC survival and enhanced functions were associated with a significant reduction of retinal inflammation, as demonstrated by significantly decreased activation of microglia and astrocytes in 1,25(OH)_2_D_3_-treated retina, compared to controls. In fact, as previously reported, activated glial cells in the retina and in the optic nerve head exert detrimental effects at different phases of glaucoma progression [46–48]. Moreover, active astrocytes are generally considered biomarkers of RGCs dysfunction [8]. Indeed, an altered crosstalk between RGCs, microglia, and astrocytes is considered an early detrimental factor in the pathophysiology of glaucoma [49]. In this context, 1,25(OH)_2_D_3_ delivered anti-inflammatory effects by decreasing the number of activated retinal microglia and astrocytes in glaucomatous mice, compared to controls. In order to confirm our experimental hypothesis and to address our molecular biology investigations on DBA/2J retinas, we carried out a third-party re-analysis of expression profiles in the optic nerve head (ONH) of DBA/2J mice with severe glaucoma, already deposited at GEO DataSet (GSE26299) [32]. In this third-party re-analysis, the 1,25(OH)_2_D_3_ receptor (VDR) was found to be overexpressed in aged DBA/2J mice, possibly due to a counter-regulatory effect, related to dysregulated signalling of 1,25(OH)_2_D_3_ [50]. As regards cytokine/chemokine expression, we found in re-analysis of GSE26299 that IL-1β and CCL-3 were upregulated in comparison to control mice (non-glaucomatous mice). On the other hand, the GSE26299 re-analysis highlighted a reduced expression of neuroprotective factors (BDNF, VEGF-A, and PlGF) in the ONH of glaucomatous mice, compared to controls.

On the basis of third-party re-analysis of GSE26299, we analysed, in our experimental setting, the retinal levels of activated NF-κB, which promotes the transcription of inflammatory cytokines [8]. In turn, activated NF-κB and released cytokines can trigger the activation of microglia and astrocytes, then amplifying the retinal immune response [48]. According to our experimental hypothesis, we found that 1,25(OH)_2_D_3_ led to a significant reduction in p65-NF-κB phosphorylation, with the subsequent reduction of pro-inflammatory cytokines and chemokines expression. Indeed, our qRT-PCR data highlighted significant (*p* < 0.05) decreased mRNA levels of IL-1β, IL-6, IFN-γ, and CCL-3 in retinas of 1,25(OH)_2_D_3_-treated mice, compared to vehicle-treated DBA/2J group. Besides its pro-inflammatory activity, IL-6 has been found to be neuroprotective on RGCs in an in vitro model of acute glaucoma: hydrostatic pressure elevation on primary RGCs [51, 52]. These intriguing results are not in contrast with our experimental and in silico data on aged glaucomatous mice. In fact, sub-chronic and chronic inflammation is detrimental for the retina, on the contrary, acute inflammatory response, through e.g., IL-6 release, is actually a first-line defence against cell damage and is necessary for healing processes [53]. As regards retinal neurotrophic factors expression, our third-party bioinformatic and experimental analyses showed that neurotrophic factors levels were reduced in glaucomatous DBA/2J mice, and 1,25(OH)_2_D_3_ treatment restored the expression of BDNF, VEGF-A, and PlGF in the retina of treated mice. Several studies reported that BDNF, VEGF-A, and PlGF are neuroprotective both in the CNS and in neuroretina [54, 55]. In particular, VEGF-A exerts protective effects against ischemic injury in retinal and brain neurons [54]. Moreover, human VEGF-A, activating ERK-1/2 and PI3K/Akt pathways, protected axotomized RGCs from degeneration and death [56]. BDNF is endowed of neuroprotective effects against various types of neuronal damages [57]. On the other hand, PlGF was reported to contribute to neuroprotection in cerebral ischemia [55]. Several reports suggested that these growth factors could be modulated by 1,25(OH)_2_D_3_ [58]. In fact, 1,25(OH)_2_D_3_ stimulates the VEGF, possibly the trophic isoform VEGF-A_165b_ and BDNF production in several types of cells [59], through the binding of VDR to the VEGF-A promoter [60]. Our results have an important translational impact, considering that hypovitaminosis D or VDR polymorphisms [61] have been found in glaucoma patients. Our post hoc bioinformatic analysis, i.e., network analysis (Fig. 9), confirmed our experimental data, evidencing that 1,25(OH)_2_D_3_ can deeply impact expression of proteins and genes involved in the etiopathogenesis of glaucoma, specifically targeting neuroinflammation through pleiotropic effects. These findings strengthen our results and the rationale of 1,25(OH)_2_D_3_ supplementation in glaucomatous patients, preferentially during early stages of the disease. Particularly, we treated mice with an intraperitoneal injection of a human equivalent dose [26] of 1,25(OH)_2_D_3_, which corresponds to the recommended daily intake, i.e., 5 μg or 200 IU per oral dose (UE regulation N. 1169/2011). This human equivalent dose was effective in our glaucoma animal model, although it was below the 1,25(OH)_2_D_3_ dose (~ 600 UI) recommended for the elderly [62], who are the main glaucomatous population. These data suggest that 1,25(OH)_2_D_3_ treatment represents a valid retinal protection strategy, warranting further clinical evaluation of the compound for the treatment of glaucoma.

## Data Availability

This study did not generate data to be deposited in external repositories. A third-party re-analysis was carried out on the GSE26299, which was correctly cited within the manuscript. The data generated during the current study are included within the article.

## Abbreviations

RGCs: Retinal ganglion cells
PERG: Pattern electroretinogram
FERG: Flash electroretinogram
IOP: Intraocular pressure
RBPMS: RNA binding protein, mRNA processing factor
Iba-1: Ionized calcium-binding adaptor protein-1
GFAP: Glial fibrillary acidic protein
NF-κB: Nuclear factor kappa-light-chain-enhancer of activated B cells
